# Characteristics of multiple Fano resonances in waveguide-coupled surface plasmon resonance sensors based on waveguide theory

**DOI:** 10.1038/s41598-018-20952-7

**Published:** 2018-02-07

**Authors:** Liu Yang, Jicheng Wang, Li-zhi Yang, Zheng-Da Hu, Xiaojun Wu, Gaige Zheng

**Affiliations:** 1 School of Science, Jiangsu Provincial Research Center of Light Industrial Optoelectronic Engineering and Technology, Jiangnan University, Wuxi, 214122 China; 2 State Key Laboratory of Millimeter Waves, Southeast University, Nanjing, 210096 China; 3 School of IoT Engineering, Jiangnan University, 214122 Wuxi, China; 4 Jiangsu Key Laboratory for Optoelectronic Detection of Atmosphere and Ocean, School of Physics and Optoelectronic Engineering, Nanjing University of Information Science and Technology, Nanjing, 210044 China

## Abstract

We observe and analyze multiple Fano resonances and the plasmon-induced transparency (PIT) arising from waveguidecoupled surface plasmon resonance in a metal-dielectric Kretschmann configuration. It is shown that the simulation results for designed structures agree well with those of the dispersion relation of waveguide theory. We demonstrate that the coupling between the surface plasmon polariton mode and multi-order planar waveguide modes leads to multiple Fano resonances and PIT. The obtained results show that the number of Fano resonances and the linewidth of resonances depend on two structural parameters, the *Parylene C* and *SiO*_2_ layers, respectively. For the sensing action of Fano resonance, the figure of merit for the sensitivity by intensity is estimated to be 44 times higher than that of conventional surface plasmon resonance sensors. Our research reveals the potential advantage of sensors with high sensitivity based on coupling between the SPP mode and multi-order PWG modes.

## Introduction

The interaction of freely oscillating electrons on a metallic surface with photons can induce surface plasmon polaritons (SPPs). These are electromagnetic waves that propagate along the surface of a metal-insulator interface^1^ and exponential evanescent field that is excited by transverse magnetic polarized light perpendicular to the interface. Transverse magnetic polarized light on a prism at an angle greater than the critical angle can generally lead to total internal reflection (TIR) at the interface of the metal and prism in a Kretschmann configuration, and evanescent waves for TIR can match with the wave vector of SPP mode to motivate SPPs. Due to the characteristics of SPPs, optical sensors and biomolecular interactions^1–4^ based on SPPs have been investigated widely. However, the metal layer of the conventional prism-based sensors causes a broad surface plasmon resonance (SPR), which can limit the sensitivity and resolution of sensors. More attention is being focused on obtaining a narrower linewidth of resonances to improve their performance in many SPR structures. SPR sensors based on long-range SPPs^5–7^ and waveguide (WG)-coupled SPR^2,3,8^ have been proposed. WG-coupled SPR sensors combining planar waveguides (PWGs) with SPR can achieve sharp resonance curves for both the p- and s-polarized incident light from the excitation of PWG modes^2,3,9^.

Hayashi *et al*. recently achieved coupling between SPPs and PWG modes^10–14^. They proposed a planar multilayer structure that exhibits plasmon-induced transparency (PIT) and Fano resonance in a Kretschmann configuration. PIT is called electromagnetically-induced transparency (EIT)^15,16^, because plasmonic effects have an EIT-like lineshape^17^ characterized by a sharp transmission band in the middle of a broad absorption band. The interference between a continuum state and a discrete level can cause Fano resonance (FR) with an asymmetric lineshape^18,19^, then the coupling between a broad and a narrow resonance can arise from so-called Fano resonance. In the past decade, people have tried to achieve EIT-like and Fano lineshapes in various nanostructures, including plasmonic nanostructures^18–30^ and metamaterials^17,31–35^. Hayashi *et al*. demonstrated experimentally and numerically the coupling between a SPP mode and a PWG mode, the reflectivity spectra, and the widths of FR and PIT lineshapes^10,11^. They also showed an almost perfect analogy between an electromagnetic (EM) system and a system of coupled oscillators (COs)^14^. Although they referred to high-order modes^11^, our research provides a detailed explanation.

In this paper, we propose a planar structure of waveguide-coupled SPR sensor, it is a metal-dielectric Kretschmann structure that can achieve multiple Fano resonances and PIT. From the analysis of electromagnetic calculations made for this structure, we illustrate that multiple Fano resonances and PIT are attributed to the coupling between the SPP mode and multi-order PWG modes. The simulation results for a designed structure agree well with those of the dispersion relation of waveguide theory. Also, we demonstrate that two structural parameters respectively govern the number of Fano resonances and the linewidth of resonances. We estimate that the figure of merit of refractive index sensing for the sensitivity by intensity is 44 times higher than that of conventional SPR sensors^14^.

## Results

**Figure 1:**
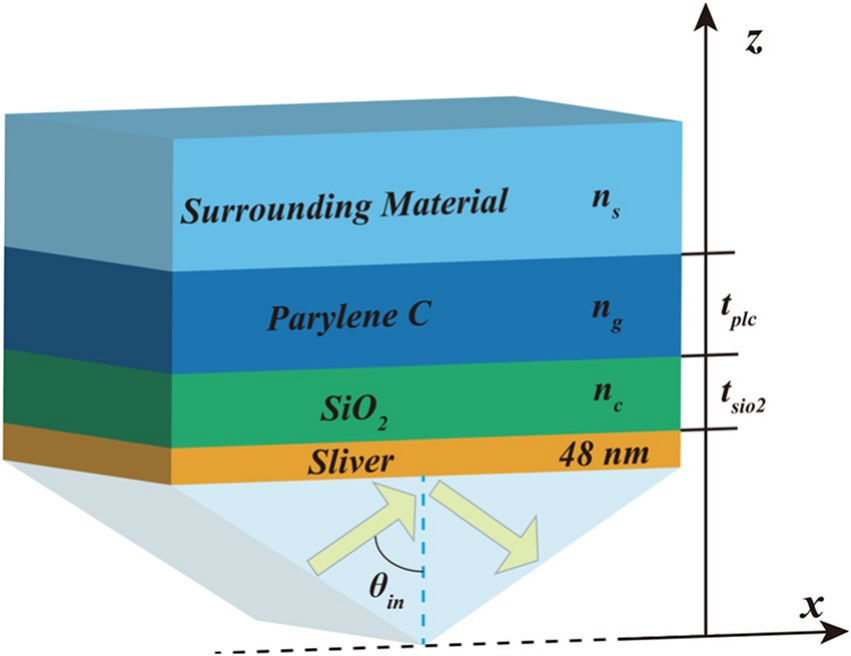
Schematic of metal-dielectric multilayer Kretschmann configuration under TM-polarized light. The refractive index of the prism is assumed as 1.7. *SiO*_2_, *Parylene C*, and the surrounding dielectric layer are placed on Ag film in sequence, and their refractive indices are fixed at *n*_*c*_ = 1.458, *n*_*g*_ = 1.62, and *n*_*s*_ = 1.333, respectively. The thicknesses of the *SiO*_2_, *Parylene C* layers are defined as ${t}_{Si{O}_{2}}$ and *t*_*plc*_, respectively.

We propose a metal-dielectric multilayer Kretschmann configuration under TM-polarized light. This consists of a prism, Ag film, a *SiO*_2_ layer, a *Parylene C* layer, and a surrounding dielectric layer, as shown in Fig. 1. The *SiO*_2_ and surrounding dielectric layer are separated by a *Parylene C* layer whose refractive index is larger than those of the *SiO*_2_ and the surrounding dielectric layers. The three dielectric layers make up a waveguide and the *Parylene C* layer can support PWG modes. We know that a structure consisting of only a semi-infinite dielectric layer adjacent to a metal layer can support SPP mode in a conventional SPR sensor. Therefore, not only SPP mode and PWG modes can be supported, but the coupling of the SPP mode and PWG modes can be achieved if the structural parameters are selected appropriately^10^. In the calculation, the dielectric function of metal is defined by the Drude model as

1 $${\varepsilon }_{Ag}(\omega )={\varepsilon }_{\infty }-\frac{{\omega }_{p}^{2}}{{\omega }^{2}+i\gamma \omega },$$

where *ε*_∞_ is the infinite frequency dielectric constant, *ω*_*p*_ is the bulk plasma frequency, *ω* is the angular frequency, and *γ* is the collision frequency which is related to the dissipation loss in the metal. These parameters are set as 6.0, 1.5 × 10^16^ rad/s, and 7.73 × 10^13^ rad/s, respectively^36^.

**Figure 2:**
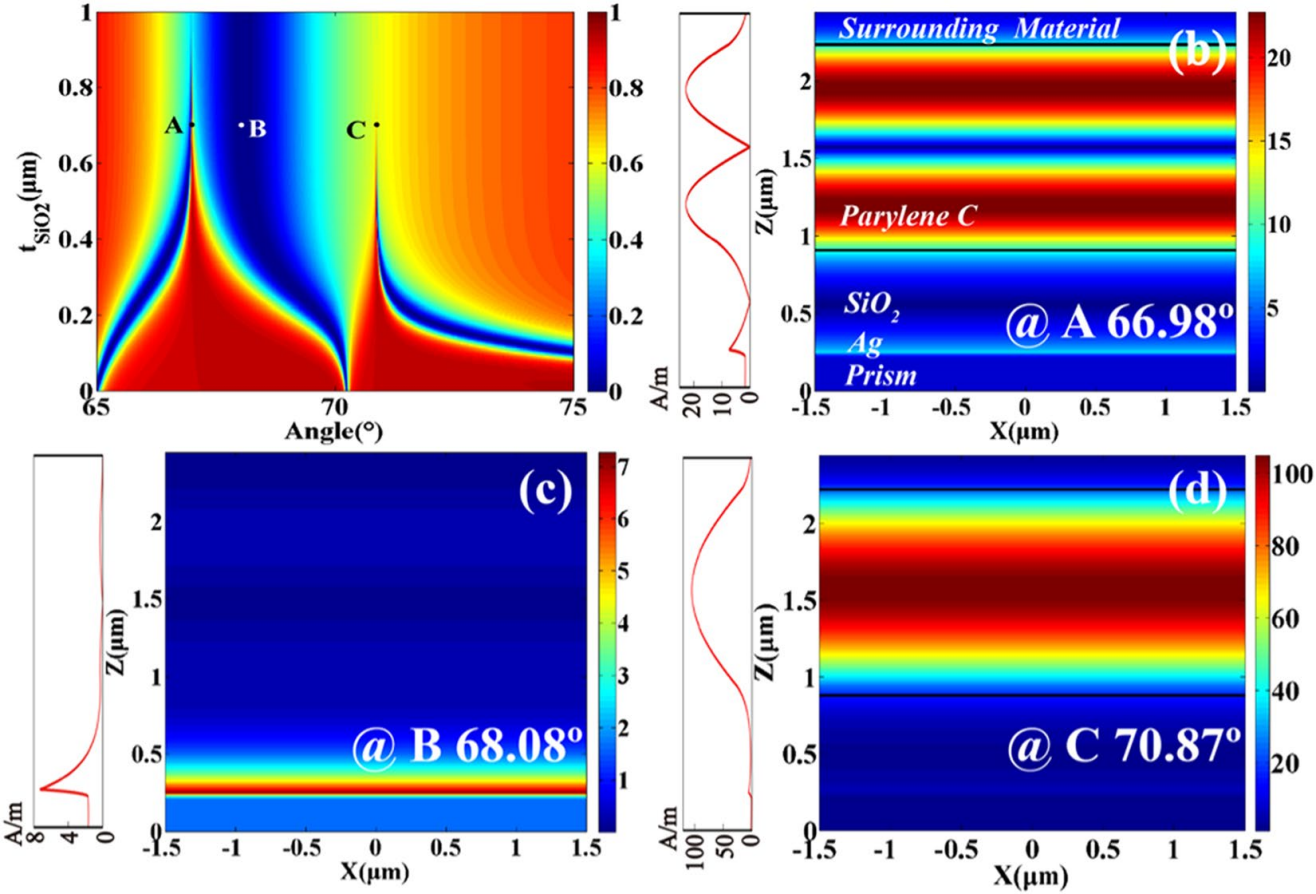
The thickness of *Parylene C* layer *t*_*plc*_ = 1.3 *μm*, *n*_*c*_ = 1.458, *n*_*g*_ = 1.62, *n*_*s*_ = 1.333. (**a**) Contour plots of the reflection versus incident angle and the thickness of the *SiO*_2_ layer. (**b**–**d**) Distributions of the magnetic field and field intensity curves are plotted at the three points (the three points are under different angles and ${t}_{Si{O}_{2}}={\rm{0.7}}$ *μm*), as shown in Fig. 2(a). The division of the structure area is shown in Fig. 2(b), and the material between two black lines is *Parylene C* layer.

Figure 2(a) shows the map of reflection spectra calculated at different angles under TM-polarized light with a wavelength of 632.8 *μ*m where t_*plc*_ is assumed to 1.3 *μ*m and ${{\rm{t}}}_{Si{O}_{2}}$ is changed from 0 to 1 *μ*m. Figure 2(b,c and d) show the distributions of the magnetic field and field intensity curves corresponding to the three points defined as A, B, and C, respectively. The magnetic field of A is not only concentrated within the *Parylene C* layer and there is one field node throughout the whole map of reflection spectra, but also is focused on the Ag − *SiO*_2_ interface, as shown in Fig. 2(b). It is expected that the SPP mode at the interface of Ag film and *SiO*_2_ layer can achieve partial coupling with PWG modes supported in *Parylene C* layer, which can lead to a Fano resonance. Figure 2(c) shows that the magnetic field is strong at the interface of Ag film and the *SiO*_2_ layer, which indicates excitation of the SPP mode (corresponding to B). Figure 2(d) shows that the strong and almost complete coupling between the SPP mode and PWG modes can be observed in the *Parylene C* layer, which can also lead to a sharp Fano resonance. From the distributions of the magnetic field, if the Fano resonance is far from the broad SPP resonance (corresponding to C), the magnetic field on the Ag − *SiO*_2_ interface is weaker, indicating that the degree of coupling between the SPP mode and PWG modes is strong. Conversely, if the Fano resonance is close to the broad SPP resonance (corresponding to A), the magnetic field on the Ag − *SiO*_2_ interface is stronger, indicating that the degree of the coupling between SPP mode and PWG modes is weak. Here, we can conclude that the distributions of the magnetic field can reflect the degree of coupling between the SPP mode and PWG modes.

**Figure 3:**
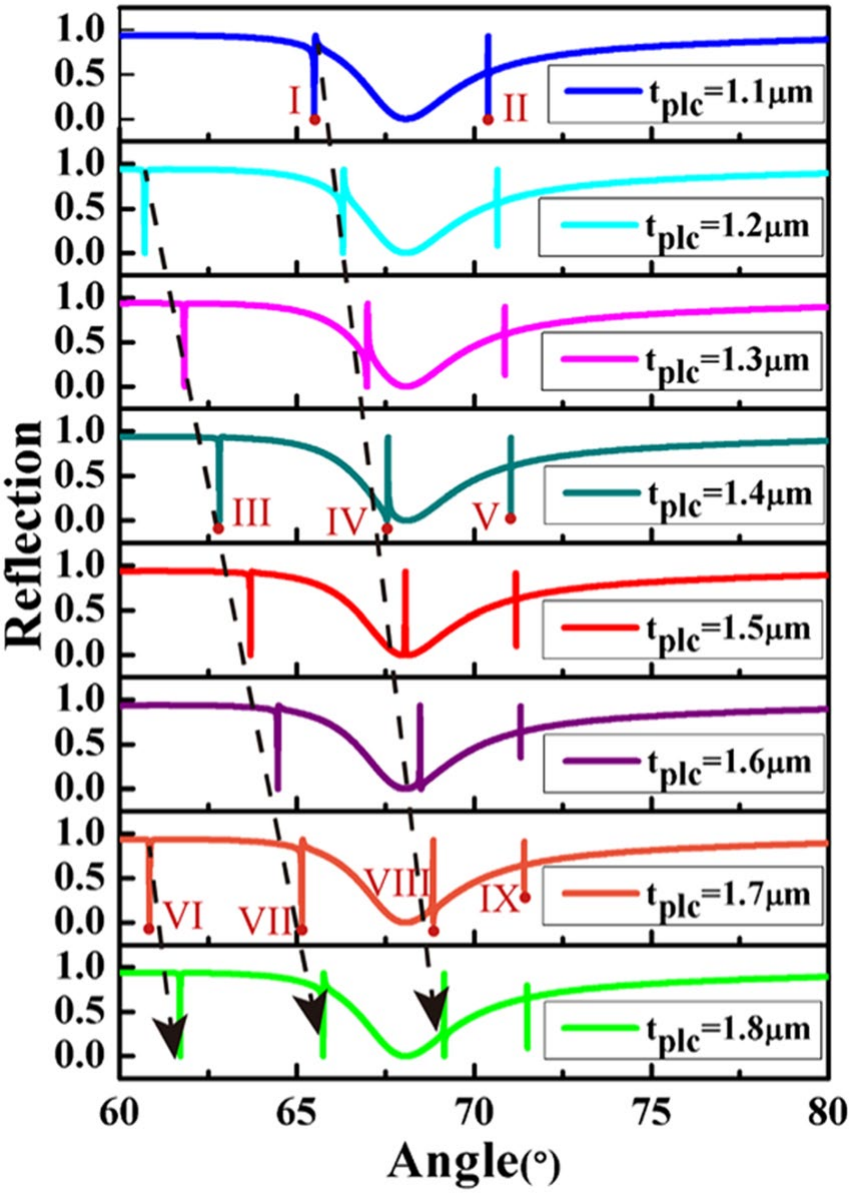
Reflection curves plotted as a function of incident angle with *t*_*plc*_ ranging from 1.1 to 1.8 *μ*m. The dashed lines indicate the position shift of multiple Fano resonances. The thickness of *SiO*_2_ layer ${t}_{Si{O}_{2}}={\rm{0.7}}$ *μm*, *n*_*c*_ = 1.458, *n*_*g*_ = 1.62, *n*_*s*_ = 1.333.

**Figure 4:**
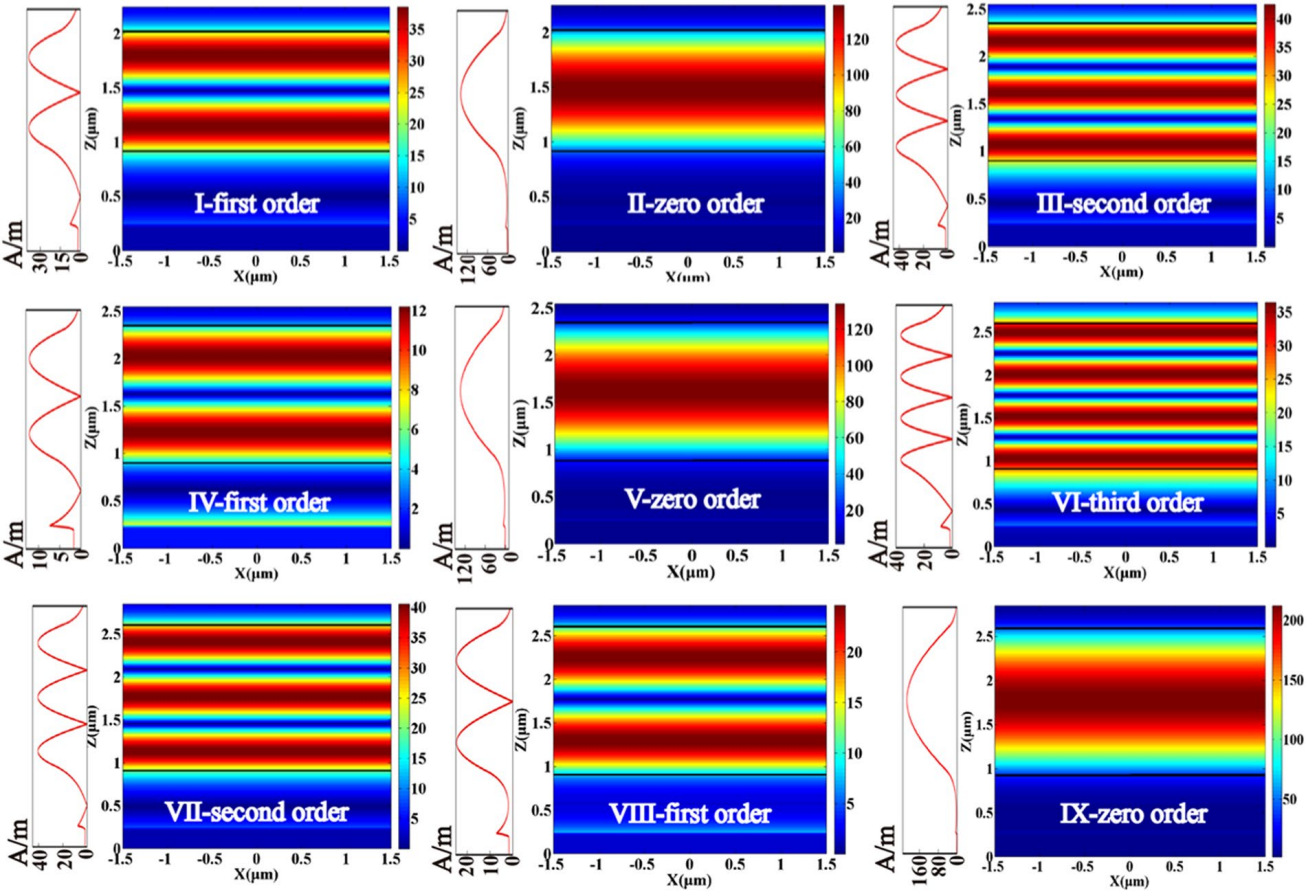
Distributions of the magnetic field and field intensity curves are plotted to present the variation tendency of SPP mode and different-order PWG modes at these points as shown in Fig. 3. The other parameters used are the same as those in Fig. 3. The material between two black lines is *Parylene C* layer.

When ${t}_{Si{O}_{2}}$ is fixed at 0.7 *μ*m, the reflectivity calculated under TM polarization light with a wavelength of 632.8 *μ*m for the proposed structure shows that the Fano resonances have a shift and the number of Fano resonances increases with the change of _*tplc*_ from 1.1 *μ*m to 1.8 *μ*m, as shown in Fig. 3, while for *t*_*plc*_ = 1.5 *μm*, a typical lineshape of PIT appears. The distributions of the magnetic field and field intensity curves of these points marked in Fig. 3 are shown in Fig. 4. When *t*_*plc*_ is fixed at 1.1 *μ*m, two Fano resonances, I and II, correspond to the magnetic field distributions I-first order and II-zero order, respectively, in Fig. 4. Similarly, the three Fano resonances III, IV, and V with *t*_*plc*_ = 1.4 *μm* correspond to the magnetic field distributions III-second order, IV-first order, and V-zero order, and four Fano resonances VI, VII, VIII, and IX with *t*_*plc*_ = 1.7 *μm* correspond to the magnetic field distributions VI-third order, VII-second order, VIII-first order, and IX-zero order, respectively. It is clear that the magnetic field distributions of zero-order Fano resonances are concentrated within the PWG layer and have no field node, which is attributed to the complete coupling of the SPP mode and TM_0_ PWG modes. For the magnetic field distributions of first-, second-, and third-order Fano resonances, the magnetic fields exist in the Ag − *SiO*_2_ interface and PWG layer with one, two and three field nodes, respectively, which respectively depend on the partial coupling between the SPP mode and the TM_1_, TM_2_, and TM_3_ PWG modes. Therefore, the positions of Fano resonances move with an increase in the number of Fano resonances. We can also conclude that the distributions of the magnetic field are used to observe the coupling between the SPP mode and different-order PWG modes. Note that we do not show all simulated Fano resonances in reflectivity curves. We can use a part of the simulated Fano resonances to explicitly illustrate that multiple Fano resonances are attributed to the coupling between the SPP mode and multi-order PWG modes.

**Figure 5:**
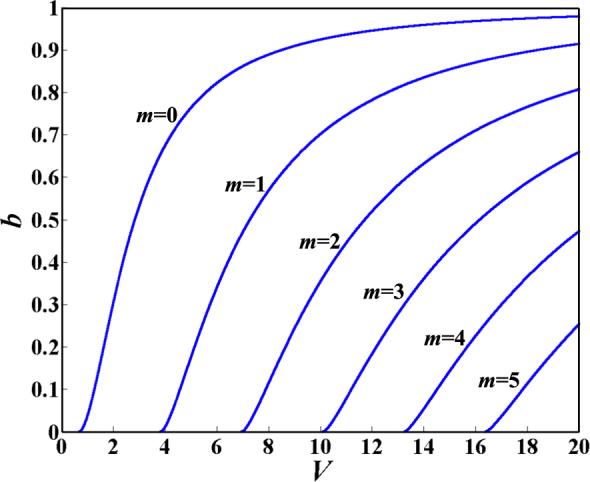
Plots of *b* versus *V* for TM modes with a wavelength of 632.8 *μ*m calculated by equation 21. The parameters used in equation 21 are *n*_*g*_ = 1.62, *n*_*s*_ = 1.333, *n*_*c*_ = 1.458.

We turn to theory to further clarify why the number of Fano resonances increases and Fano resonances shift with the increase of *t*_*plc*_. According to the dispersion relation of waveguides^37^, the plots of the generalized guide index *b* versus the generalized frequency *V* for TM modes with parameters of the designed structure are depicted in Fig. 5. We can also obtain the cutoff *V* and cutoff thickness of the waveguide *h* in a designed structure corresponding to each mode, as shown in Table 1. Although Hayashi *et al*. refer to high-order modes^11^ refer to high-order modes, they provide no explanation for their appearance. We will theoretically analyze the reasons for the appearance of high-order PWG modes.

**Table 1:** Cutoff *V* and cutoff thickness *h* corresponding to each mode. The parameters used in equations 22–23 are the same as those in Fig. 5.

Guide mode	Cutoff *V*	Cutoff thickness *h* (*μm*)
*m* = 0	*V* = 0.6699	*h* = 0.073287
*m* = 1	*V* = 3.8115	*h* = 0.41697
*m* = 2	*V* = 6.9531	*h* = 0.76066
*m* = 3	*V* = 10.095	*h* = 1.1043
*m* = 4	*V* = 13.236	*h* = 1.448
*m* = 5	*V* = 16.378	*h* = 1.7917

**Figure 6:**
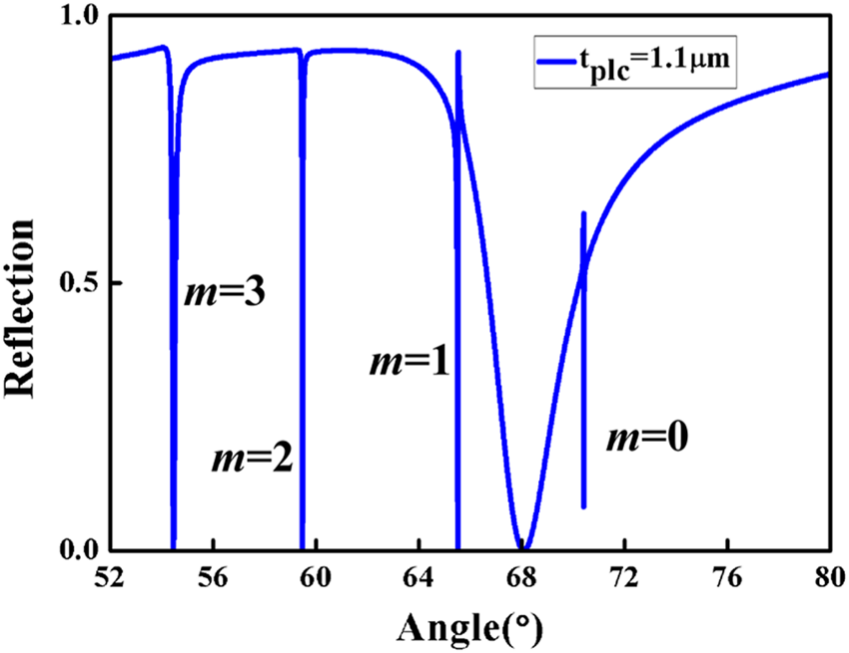
Reflection curve is plotted as a function of incident angle. The thickness of the *Parylene C* layer is *t*_*plc*_ = 1.1 *μm*, the thickness of the *SiO*_2_ layer is ${t}_{Si{O}_{2}}={\rm{0.7}}$ *μm*, *n*_*c*_ = 1.458, *n*_*g*_ = 1.62, *n*_*s*_ = 1.333.

In Fig. 5 and Table 1, the generalized frequency *V* is called as the cutoff *V* when the generalized guide index *b* is equal to zero. It is also obvious that a kind of TM_*m*_ (*m* = 0, 1, 2, 3 ⋅⋅⋅) PWG mode can be supported by a waveguide if the generalized frequency *V* is equal to or larger than the cutoff *V* (or the waveguide *h* is equal to or larger than the cutoff *h*). Therefore, from Fig. 5 and Table 1, we can surmise that the waveguide in the proposed structure can support multi-order PWG modes with *t*_*plc*_ ranging from 1.1 to 1.8 *μ*m. Under the appropriate structural parameters, the SPP mode can couple with different orders of PWG modes, which leads to multiple Fano resonances. In a fixed range, the number of Fano resonances increases, while the positions of Fano resonances move with it. For example, when *t*_*plc*_ = 1.1 *μm* in simulation, there are four Fano resonances, as shown in Fig. 6, which suggests that the SPP mode couples with four orders of PWG modes. In theory, the thickness of the *Parylene C t*_*plc*_ is approximately equal to the cutoff thickness 1.1043 *μ*m from Table 1, so four orders of PWG modes can be supported in a waveguide layer, and the coupling between the SPP mode and four orders of PWG modes can lead to four Fano resonances. It is clear that the simulation results for the designed structure agree well with those of the dispersion relation of waveguide theory.

**Figure 7:**
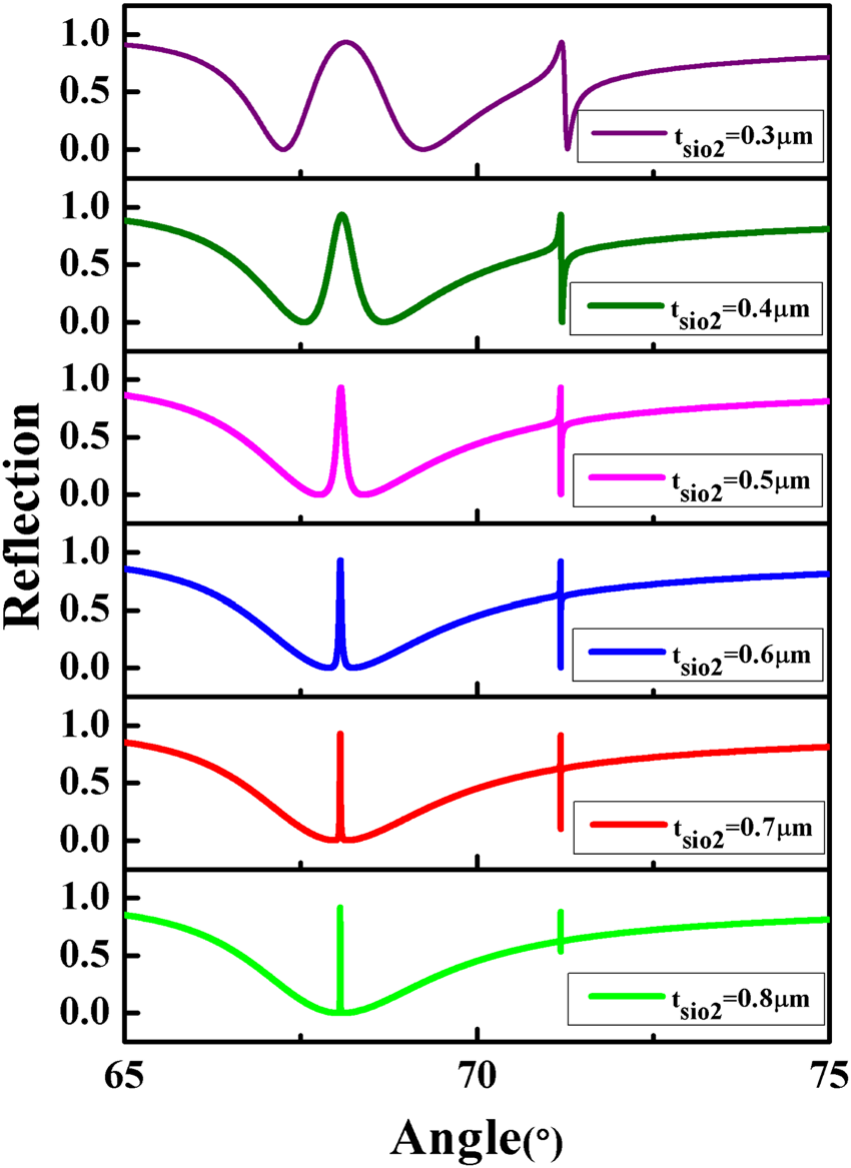
Reflection curves are plotted as a function of incident angle with ${t}_{Si{O}_{2}}$ ranging from 0.3 to 0.8 *μ*m. The thickness of the *Parylene C* layer is *t*_*plc*_ = 1.5 *μm*, *n*_*c*_ = 1.458, *n*_*g*_ = 1.62, *n*_*s*_ = 1.333.

From Fig. 3, we can see that a typical lineshape of PIT appears with *t*_*plc*_ = 1.5 *μm*. Therefore, we discuss the effect of the thickness of *SiO*_2_ on the lineshape of PIT, when *t*_*plc*_ is 1.5 *μ*m. Figure 7 shows the reflection spectra with different thicknesses of *SiO*_2_ under TM polarization light with a wavelength of 632.8 *μ*m. It is clear that the linewidth of PIT is increasingly narrow as the thickness of *SiO*_2_ increases. Also, the linewidth of Fano resonance on the right side of the PIT shows a similar change, and the amplitude of Fano resonance can be affected by the thickness of *SiO*_2_.

**Figure 8:**
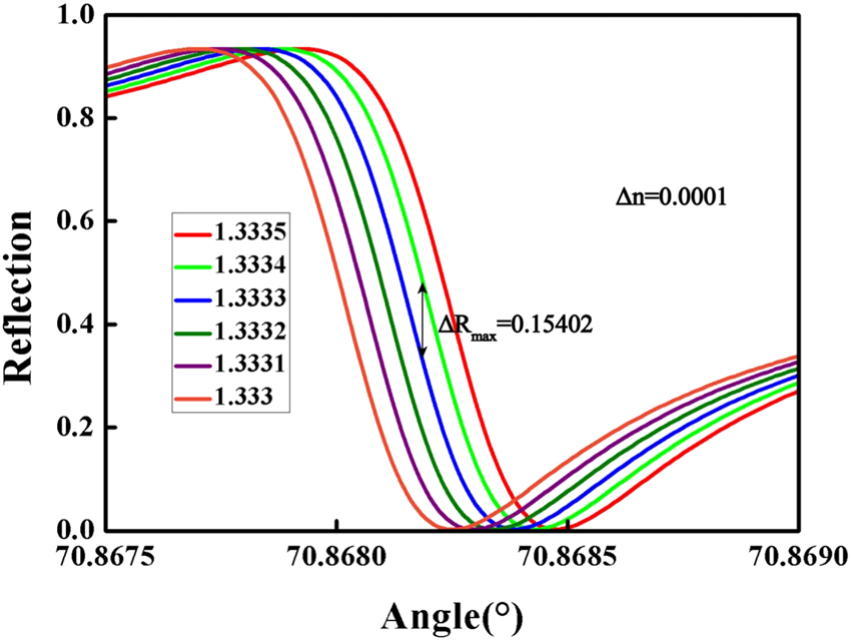
Shift of TM_0_ Fano resonance for the structure with ${t}_{Si{O}_{2}}={\rm{0.7}}$ *μm* and *t*_*plc*_ = 1.3 *μm*. The refractive index of surrounding material changes from 1.333 to 1.3335 with steps of Δ*n* = 0.0001. *n*_*c*_ = 1.458, *n*_*g*_ = 1.62.

The shift of TM_0_ Fano resonance curves, which can be considered the sensitivity for sensors, is caused by a change in the refractive index (RI) of surrounding material.It is shown in Fig. 8. To compare with the performance of the conventional SPR sensors, we usually use either an angular shift of the Δ*θ*_*res*_ curve (sensing by angular modulation) or a change in the reflectance Δ*R* at a fixed angle (sensing by intensity modulation) to describe the change in the resonance curve caused by a change in the RI Δ*n*^4,38^. The sensitivity by intensity is given by

2 $${S}_{I}(\theta )=\mathop{\mathrm{lim}}\limits_{{\rm{\Delta }}n\to 0}\frac{{\rm{\Delta }}R}{{\rm{\Delta }}n}=\frac{\partial R(\theta )}{\partial n}\mathrm{.}$$

It is convenient to compare the sensitivities of different types of sensors by using the figure of merit for sensitivity by intensity, given by

3 $$FO{M}_{I}=\mathop{{\rm{\max }}}\limits_{\theta }|{S}_{I}(\theta )|,$$

which is the maximum value of the sensitivity by intensity. For a conventional SPR sensor, it consists of a 50 nm-thick Au film deposited on a SF10 prism, the SPP resonance in the conventional SPR sensor is broad and has a small slope. Therefore, Δ*n* as small as 1 × 10^−2^ is required to produce a change in the reflectance of ${\rm{\Delta }}{R}_{\max }=\mathrm{\ 0.35}$ the ratio ${\rm{\Delta }}{R}_{\max }/{\rm{\Delta }}n=35{{\rm{RIU}}}^{-1}$ ^14^. However, the present TM_0_ Fano sensor can produce the change ${\rm{\Delta }}{R}_{\max }=0.15402$ (the maximum value in the Fig. 8, when Δ*n* is as small as 1 × 10^−4^. The ratio ${\rm{\Delta }}{R}_{\max }/{\rm{\Delta }}n$ is 1540.2 RIU^−1^. These values suggest that the present Fano sensor has an extremely high sensitivity by intensity compared to that of the conventional SPR sensor. It is estimated that *FOM*_*I*_ of the present TM_0_ Fano sensor is at least 44 times than that of the conventional SPR sensor.

## Discussion

In conclusion, we propose a metal-dielectric multilayer Kretschmann structure that can achieve multiple Fano resonances and PIT resulting from the coupling between the SPP mode and multi-order PWG modes. We conclude that the coupling between the SPP mode and multi-order PWG modes can lead to multiple Fano resonances from electromagnetic calculations. It is important that the calculations of the dispersion relation of waveguide theory are consistent with those of the designed structure. Also, we observe that two structural parameters, the thicknesses of *SiO*_2_ and *Parylene C*, influence the number of Fano resonances and the linewidth of resonances, respectively. We also analyze the sensing action of the proposed TM_0_ Fano resonance. Its the figure of merit for the sensitivity by intensity is 44 times greater than that of a conventional SPR sensor. Our results may pave the way in the coupling between the SPP mode and multi-order PWG modes and for the design of efficient sensors with high sensitivity.

## Methods

A TM mode has a magnetic field component, *h*_*y*_ and two electric field components, *e*_*x*_ and *e*_*z*_. The transverse electric field component *e*_*x*_ is normal to the waveguide surface and the direction of propagation. In addition, the two electric field components can be expressed in terms of *h*_*y*_. Specifically, we obtain^37^

4 $${e}_{x}(x)=\frac{\beta }{\omega {\varepsilon }_{0}{n}^{2}}{h}_{y}(x)$$

5 $${e}_{z}(x)=-j\frac{1}{\omega {\varepsilon }_{0}{n}^{2}}\frac{d{h}_{y}(x)}{dx}\mathrm{.}$$

The boundary conditions for *h*_*y*_, *e*_*z*_, and *e*_*x*_ are met if *h*_*y*_ and (1/*n*^2^)/(*dh*_*y*_/*dx*) are continuous at the boundaries. We write *h*_*y*_ in the cover, film and substrate regions as

6 $${h}_{y}(x)={H}_{c}{e}^{-{\gamma }_{c}x}$$

7 $${h}_{y}(x)={H}_{f}\,\cos ({k}_{f}x+\phi ^{\prime} )$$

8 $${h}_{y}(x)={H}_{s}{e}^{{\gamma }_{s}(x+h)},$$

where *H*_*c*_, *H*_*f*_, *H*_*s*_, and *φ*′ are constants to be determined. By matching the boundary values, we obtain the dispersion relation or characteristic equation for TM modes:

9 $${k}_{f}h={\tan }^{-1}\frac{{n}_{f}^{2}}{{n}_{c}^{2}}\frac{{\gamma }_{c}}{{k}_{f}}+{\tan }^{-1}\frac{{n}_{f}^{2}}{{n}_{s}^{2}}\frac{{\gamma }_{s}}{{k}_{f}}+m\pi ,$$

where

10 $${k}_{f}=\sqrt{{k}^{2}{n}_{f}^{2}-{\beta }^{2}}$$

11 $${\gamma }_{s}=\sqrt{{\beta }^{2}-{k}^{2}{n}_{s}^{2}}$$

12 $${\gamma }_{c}=\sqrt{{\beta }^{2}-{k}^{2}{n}_{c}^{2}}\mathrm{.}$$

The mode number *m* is an integer. When *β* and three of the four constants are determined, the TM problem is solved. The fourth constant, say *H*_*f*_ or *H*_*c*_, represents the amplitude of the TM mode. In particular, *H*_*c*_ is the magnetic field intensity at the cover-film boundary and *H*_*f*_ is the peak magnetic field intensity of the TM mode in question. To circumvent the difficulties in the calculation process, Kogelnik and Ramaswany introduced generalized parameters^39^.

Several generalized parameters^39–41^ can describe TM modes guided by three-layer step-index waveguides. These generalized parameters are

- the asymmetry measure,
  13 $$a=\frac{{n}_{s}^{2}-{n}_{c}^{2}}{{n}_{f}^{2}-{n}_{s}^{2}}$$
- the generalized frequency, also known as the generalized film thickness,
  14 $$V=kh\sqrt{{n}_{f}^{2}-{n}_{s}^{2}}$$
- the generalize guide index,

15 $$b=\frac{{N}^{2}-{n}_{s}^{2}}{{n}_{f}^{2}-{n}_{s}^{2}}$$

The generalized parameters *a* and *b* are the differences ${n}_{s}^{2}-{n}_{c}^{2}$ and ${N}^{2}-{n}_{s}^{2}$ normalized with respect to ${n}_{f}^{2}$ − ${n}_{s}^{2}$. In other words, these generalized parameters are in terms of the differences of squared indices rather than the indices themselves.

Some manipulation will show that

16 $${k}_{f}h=kh\sqrt{{n}_{f}^{2}-{N}^{2}}=V\sqrt{1-b}$$

17 $${\gamma }_{s}h=kh\sqrt{{N}^{2}-{n}_{s}^{2}}=V\sqrt{b},$$

and

18 $${\gamma }_{c}h=kh\sqrt{{N}^{2}-{n}_{c}^{2}}=V\sqrt{a+b}\mathrm{.}$$

The extra parameter can be either

19 $$c=\frac{{n}_{s}^{2}}{{n}_{f}^{2}}$$

or

20 $$d=\frac{{n}_{c}^{2}}{{n}_{f}^{2}}=c-a(1-c)\mathrm{.}$$

Using these expressions in equation 9, we can obtain the dispersion relation for TM modes. In terms of generalized parameters, we have

21 $$V\sqrt{1-b}={\tan }^{-1}\frac{1}{d}\sqrt{\frac{a+b}{1-b}}+{\tan }^{-1}\frac{1}{c}\sqrt{\frac{b}{1-b}}+m\pi \mathrm{.}$$

For a given waveguide operating at a specific wavelength, the values of *nf*, *n*_*s*_, *n*_*c*_, *h*, and *λ* are known. The values of *a*, *c*, *d*, and *V* can be calculated from the waveguide parameters. For each set of *a*, *V* and *c*, we determine *b* numerically from equation 21 for TM modes. There may be one or more solutions for *b*, depending on *V*, *a*, and *c*. Each solution for *b* corresponds to a guided mode. The largest value of *b* corresponds to *m* = 0.

As noted earlier, each solution of *b* corresponds to a guided mode. As the film thickness decreases, corresponding to a smaller *V*, *b* becomes smaller. As *b* of a given mode approaches zero, the mode approaches its cutoff. As noted previously, the cutoff condition is *b* = 0. By setting *b* to zero, we obtain, from equation 21 that the cutoff *V* for the TM_*m*_ mode is

22 $$V=m\pi +{\tan }^{-1}(\frac{\sqrt{a}}{d})=m\pi +{\tan }^{-1}(\frac{{n}_{f}^{2}}{{n}_{c}^{2}}\sqrt{a})\mathrm{.}$$

In other words, a TM_*m*_ mode is supported by a thin-film waveguide if the film thickness is at least

23 $$h=\frac{m\pi +{\tan }^{-1}(\frac{{n}_{f}^{2}}{{n}_{c}^{2}}\sqrt{a})}{2\pi \sqrt{{n}_{f}^{2}-{n}_{s}^{2}}}\lambda \mathrm{.}$$

### Fabrication process of the proposed structure

**Figure 9:**
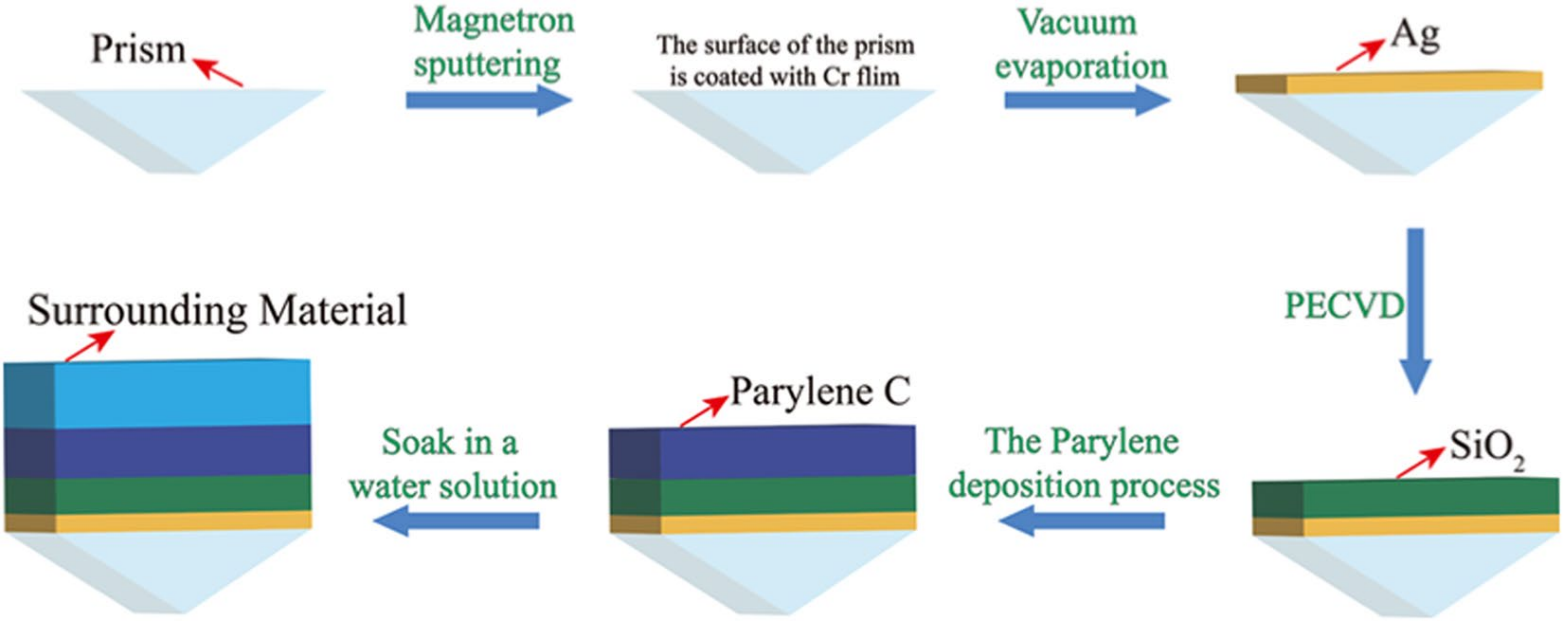
Process flow diagram of the proposed device fabrication.

Figure 9 shows a design scheme of the fabrication process for the proposed structure. First, the surface of the prism is coated with 5 nm of Cr film to plate a metal film using the magnetron sputtering method, which is one of physical vapor deposition (PVD). Next, the Ag layer is deposited with an electron-beam evaporator system. The *SiO*_2_ is then deposited using the plasma enhanced chemical vapor deposition (PECVD) method. *Parylene C* can then be deposited by the *Parylene* deposition process. Finally, the whole structure is soaked in a water solution that is a sensing medium.

### Numerical simulation

Rigorous couple wave analysis (RCWA) method simulations are performed to obtain the contour plots of the reflection, magnetic field distributions, and reflection spectra. All simulated figures are drawn using MATLAB software after processing the data, including plots of the generalized guide index *b* versus the generalized frequency *V* for TM modes, according to the dispersion relation of waveguides.
